# Diffuse Alveolar Hemorrhage in Systemic Lupus Erythematosus: An Updated Clinical Perspective on a High-Mortality Complication

**DOI:** 10.7759/cureus.107587

**Published:** 2026-04-23

**Authors:** Juan Camilo Santacruz, Marta Juliana Mantilla, Sandra Pulido, Carlos Agudelo, Julián Alberto Naranjo, Oscar Vicente Vergara, John Londono

**Affiliations:** 1 Rheumatology, Medicarte IPS, Rionegro, COL; 2 Rheumatology, Centro de Investigación en Reumatología y Especialidades Médicas (CIREEM), Bogotá, COL; 3 Rheumatology, Colsanitas, Bogotá, COL; 4 Rheumatology, Clínica Las Américas, Medellín, COL; 5 Rheumatology, ART Médica IPS, Medellín, COL; 6 Spondyloarthritis Research Group, Universidad de La Sabana, Chía, COL

**Keywords:** bronchoalveolar lavage, diffuse alveolar hemorrhage, immunosuppressive agents, pulmonary hemorrhage, systemic lupus erythematosus

## Abstract

Diffuse alveolar hemorrhage (DAH) is a rare but devastating complication of systemic lupus erythematosus (SLE), often presenting with acute respiratory failure and carrying high mortality. Clinical recognition is challenging, as hemoptysis may be absent, and early features can overlap with infection or other causes of acute respiratory distress syndrome (ARDS).

Diagnosis relies on a high index of suspicion, supported by imaging and bronchoalveolar lavage, while promptly excluding infectious etiologies. Antineutrophil cytoplasmic antibodies (ANCA)-associated vasculitis remains the most important differential diagnosis.

Management is largely based on observational data and requires early, aggressive immunosuppression with high-dose glucocorticoids and additional agents such as cyclophosphamide or rituximab. In refractory cases, adjunctive therapies and extracorporeal membrane oxygenation (ECMO) may be lifesaving.

Despite therapeutic advances, outcomes remain poor, underscoring the need for early recognition and timely intervention.

## Introduction and background

Diffuse alveolar hemorrhage (DAH) remains one of the most severe and life-threatening pulmonary complications in patients with systemic lupus erythematosus (SLE). Although rare, DAH is one of the most severe pulmonary complications of SLE, with reported mortality rates as high as 85.7% in some case series [1,2]. Epidemiological data highlight its rarity: one cohort reported DAH in only 19 out of 510 hospitalized SLE patients over approximately 10 years, while a large Spanish registry identified 37 cases among 4,024 patients, corresponding to a prevalence of approximately 0.9% [3,4].

In a large analysis of 9,320 hospitalizations for DAH, SLE accounted for 2.62% of cases, compared with 5.3% attributed to antineutrophil cytoplasmic antibodies (ANCA)-associated vasculitis. Both conditions predominantly affected women; however, patients with SLE were younger (mean age 47 years vs. 59 years) and exhibited a significantly higher risk of mortality (odds ratio (OR) 24.54) [5]. Although DAH may rarely represent the initial manifestation of SLE, it more commonly occurs in individuals with established disease, with a variable time to onset ranging from six months to over 14 years, and a mean duration of approximately three years in more recent reports [6].

The underlying pathophysiology of DAH in SLE is not fully elucidated. Current evidence suggests that immune complex deposition and apoptosis-driven endothelial injury play central roles, leading to capillary disruption and intra-alveolar bleeding [7]. From an immunological standpoint, anti-Ro (SSA) and anti-dsDNA antibodies are among the most frequently detected autoantibodies in affected patients. In addition, ANCA have been reported in up to 9.3% of cases, suggesting potential overlap or shared pathogenic mechanisms [8,9].

Furthermore, the presence of antiphospholipid antibodies - particularly lupus anticoagulant and anticardiolipin IgG - has been associated with an increased risk of DAH, whereas the role of anti-β2 glycoprotein I IgG remains less consistent. Notably, some studies have described triple antiphospholipid antibody positivity in these patients, raising the possibility of a higher-risk phenotype even in the absence of full criteria for antiphospholipid syndrome [10].

## Review

Pathophysiology and histology

DAH encompasses three principal histopathological patterns: pulmonary capillaritis, bland alveolar hemorrhage, and diffuse alveolar damage [11]. Pulmonary capillaritis is characterized by neutrophilic infiltration of the alveolar septa within the pulmonary interstitium, leading to capillary necrosis, disruption of the alveolar-capillary barrier, and subsequent extravasation of red blood cells into both alveolar and interstitial spaces [12]. Neutrophils frequently undergo fragmentation and pyknosis, supporting the pathogenic role of their byproducts, including reactive oxygen species and proteolytic enzymes, in mediating tissue injury. Cellular debris, along with fibrin deposition, accumulates within alveolar spaces and may be associated with fibrinoid necrosis of the interstitium [13].

In contrast, bland alveolar hemorrhage is defined by the presence of intra-alveolar bleeding in the absence of significant inflammation or structural destruction of the alveolar architecture. Although this pattern may be observed in SLE and anti-glomerular basement membrane disease, it is more commonly associated with coagulopathies [14]. Diffuse alveolar damage represents the histological hallmark of acute respiratory distress syndrome (ARDS) and is characterized by the formation of hyaline membranes lining the alveolar spaces. This pattern is etiologically heterogeneous and may result from infectious processes, cytotoxic and non-cytotoxic drug exposure, autoimmune diseases, hematopoietic stem cell transplantation, or acute lung allograft rejection, among others [15].

At the molecular level, neutrophil activation and subsequent cytolysis lead to the release of cytotoxic proteins and neutrophil extracellular traps (NETs), which contribute to the disruption of the alveolar-capillary basement membrane and facilitate red blood cell leakage into the alveolar space [16]. The precise mechanisms driving the development of pulmonary capillaritis remain incompletely understood; however, antiphospholipid antibodies have been implicated as potential contributors. Additionally, infectious triggers may play a role, particularly in the context of diffuse alveolar damage [17].

Unlike capillaritis, bland alveolar hemorrhage is often associated with a predominance of macrophage infiltration. Increased apoptosis of alveolar epithelial cells has been proposed in this setting, potentially enhancing the clearance of apoptotic debris while attenuating the inflammatory response. Alveolar macrophages demonstrate elevated myeloperoxidase expression, likely secondary to erythrophagocytosis, although a contributory role of immune complex deposition cannot be excluded [18].

**Figure 1:**
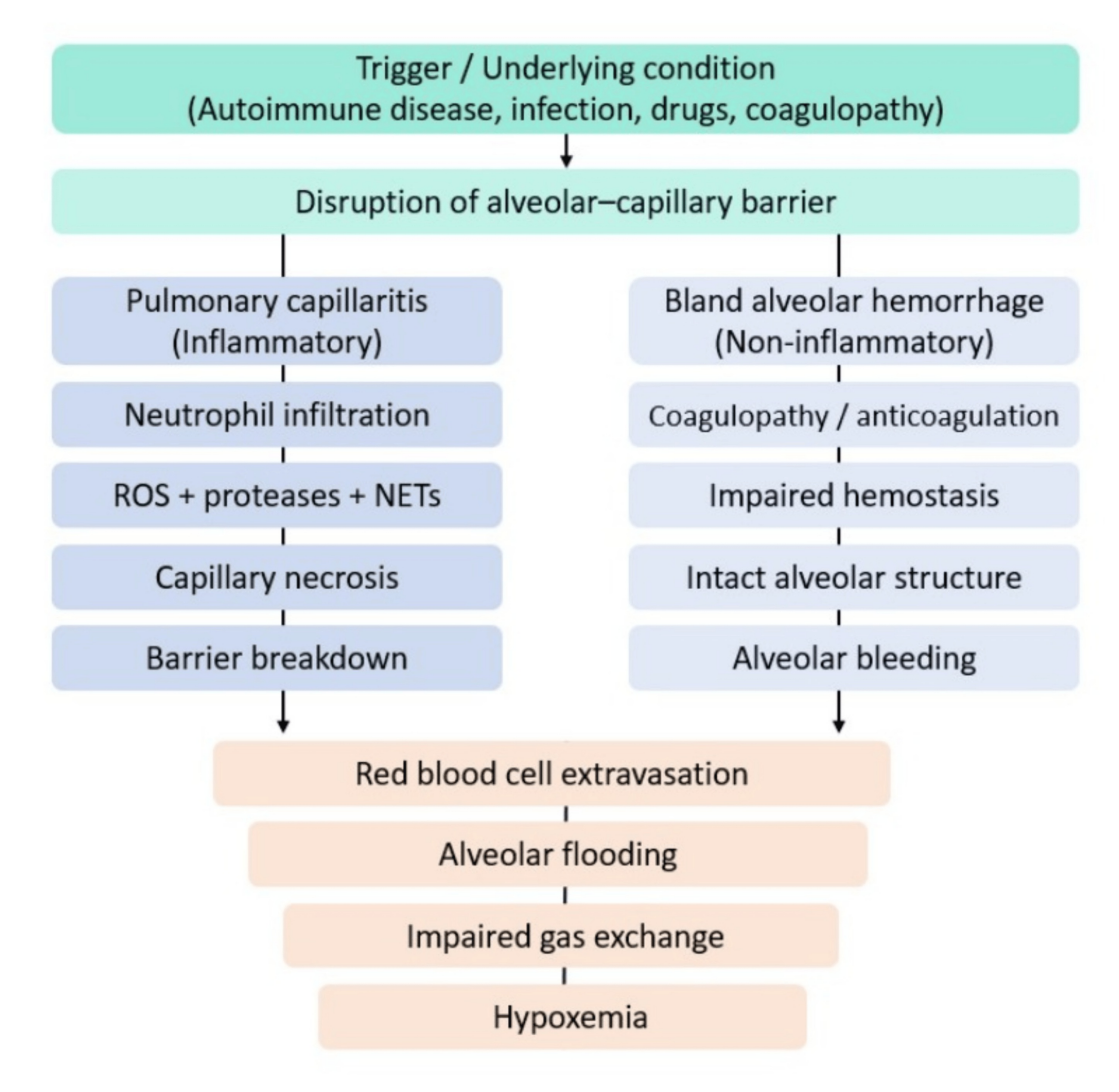
Pathophysiology of diffuse alveolar hemorrhage. Credit: Image created by the authors. NET, neutrophil extracellular trap; ROS, reactive oxygen species

The following flowchart depicts the proposed pathophysiological mechanisms of DAH (Figure 1).

Clinical manifestations

DAH in SLE typically presents as an acute and rapidly progressive clinical syndrome, most commonly characterized by dyspnea, cough, and hemoptysis, with symptom onset generally occurring within a few days [19]. Hemoptysis, although classically described, is present in only 46%-50% of cases, whereas dyspnea and hypoxemia are observed in the vast majority of patients (85.7%-100%) [20]. The classic triad of anemia, hemoptysis, and pulmonary infiltrates is identified in approximately 46.2% of cases, and its absence should not exclude the diagnosis. Fever is inconsistently reported, occurring in only about 25% of patients [21].

Alveolar bleeding is often severe enough to result in clinically significant anemia. In addition, many patients exhibit concurrent active lupus nephritis-most frequently class III or IV-along with hypocomplementemia (particularly low C3 levels), elevated anti-double-stranded DNA antibody titers, and thrombocytopenia, reflecting a state of high systemic disease activity [22,23]. Although DAH may represent the initial manifestation of SLE in some cases, it more commonly occurs in patients with an established diagnosis.

Prognostic factors associated with increased mortality include thrombocytopenia, renal impairment, the need for mechanical ventilation, and elevated Acute Physiology and Chronic Health Evaluation II (APACHE II) scores [24]. Early initiation of cyclophosphamide has shown a trend toward improved outcomes, although this has not reached statistical significance. Recurrence of DAH has been reported in up to 16% of cases in certain cohorts, underscoring the relapsing nature of this complication and the importance of sustained immunologic control through appropriate maintenance therapy [25].

Diagnosis

Flexible bronchoscopy with bronchoalveolar lavage (BAL) remains the diagnostic gold standard for DAH. Cytological analysis of BAL fluid typically reveals hemosiderin-laden macrophages, with a threshold of ≥20% considered suggestive of DAH; however, these findings may not be evident until 48-72 hours after the onset of bleeding [26]. Hemosiderin-laden macrophages are identified in approximately 67% of cases and are often accompanied by neutrophilic alveolitis. Importantly, BAL also plays a critical role in excluding infectious etiologies before the escalation of immunosuppressive therapy [27].

Chest computed tomography (CT) commonly demonstrates bilateral, diffuse ground-glass opacities and/or consolidations, although unilateral involvement may occasionally be observed. These opacities typically exhibit a central predominance rather than a peripheral distribution [28]. Notably, up to 31.5% of patients with DAH have a concomitant pulmonary infection, with bacterial pathogens being the most frequent (62%), followed by fungal (21%) and viral infections (17%) [29].

In patients with SLE and concomitant pulmonary hypertension, opportunistic infections such as cytomegalovirus (CMV) and Pneumocystis jirovecii (PJP) have been associated with significantly increased mortality at 90 and 180 days (relative risks of 5.94 and 7.13, respectively), despite appropriate treatment. Furthermore, these infections represent independent predictors of mortality (odds ratios of 14.2 and 25.5), while additional factors, including prolonged mechanical ventilation (>14 days; OR 11.1) and the use of aggressive immunosuppression near the onset of pulmonary involvement, have also been linked to worse outcomes [30].

Treatment

Recommendations regarding the optimal management of DAH in SLE have not been established through randomized controlled trials. Given its potentially life-threatening nature, aggressive treatment is warranted, typically consisting of high-dose glucocorticoids-either intravenous methylprednisolone pulses (500 mg to 1 g daily for 3-5 days) in patients with acute clinical onset, or oral prednisone at doses of 1-2 mg/kg/day in more clinically stable patients-combined with an additional immunosuppressive agent (cyclophosphamide, rituximab, azathioprine, or mycophenolate mofetil) [31].

The choice of the second immunosuppressive agent should be individualized based on disease severity, particularly in the presence of severe hypoxemia or the need for mechanical ventilation, and the extent of multiorgan involvement. In this context, cyclophosphamide is often considered the first-line option when a rapid therapeutic effect is required [32]. In patients with less severe clinical presentations, mycophenolate mofetil or azathioprine may represent reasonable alternatives, provided that close monitoring is ensured to promptly identify clinical deterioration or lack of response.

Adjunctive therapies, such as plasmapheresis and intravenous immunoglobulin (IVIG), have been used in refractory cases; however, the evidence supporting their use remains limited [33]. Recent recommendations from the American College of Rheumatology (ACR) suggest that biological agents, including belimumab and anifrolumab, may offer benefit in selected cases. Nevertheless, these recommendations evidence supporting their use remains limited, and their role in the management of DAH remains uncertain [34].

Notably, a case of SLE-associated recurrent and multi-refractory DAH has been reported, in which, following failure of multiple lines of immunosuppressive therapy, the initiation of anifrolumab was associated with sustained clinical control, resolution of alveolar bleeding, and absence of further recurrences during follow-up. Despite these promising findings, the available evidence is limited to isolated reports, and thus, the role of anifrolumab in DAH requires further validation in prospective studies [35]. 

**Figure 2:**
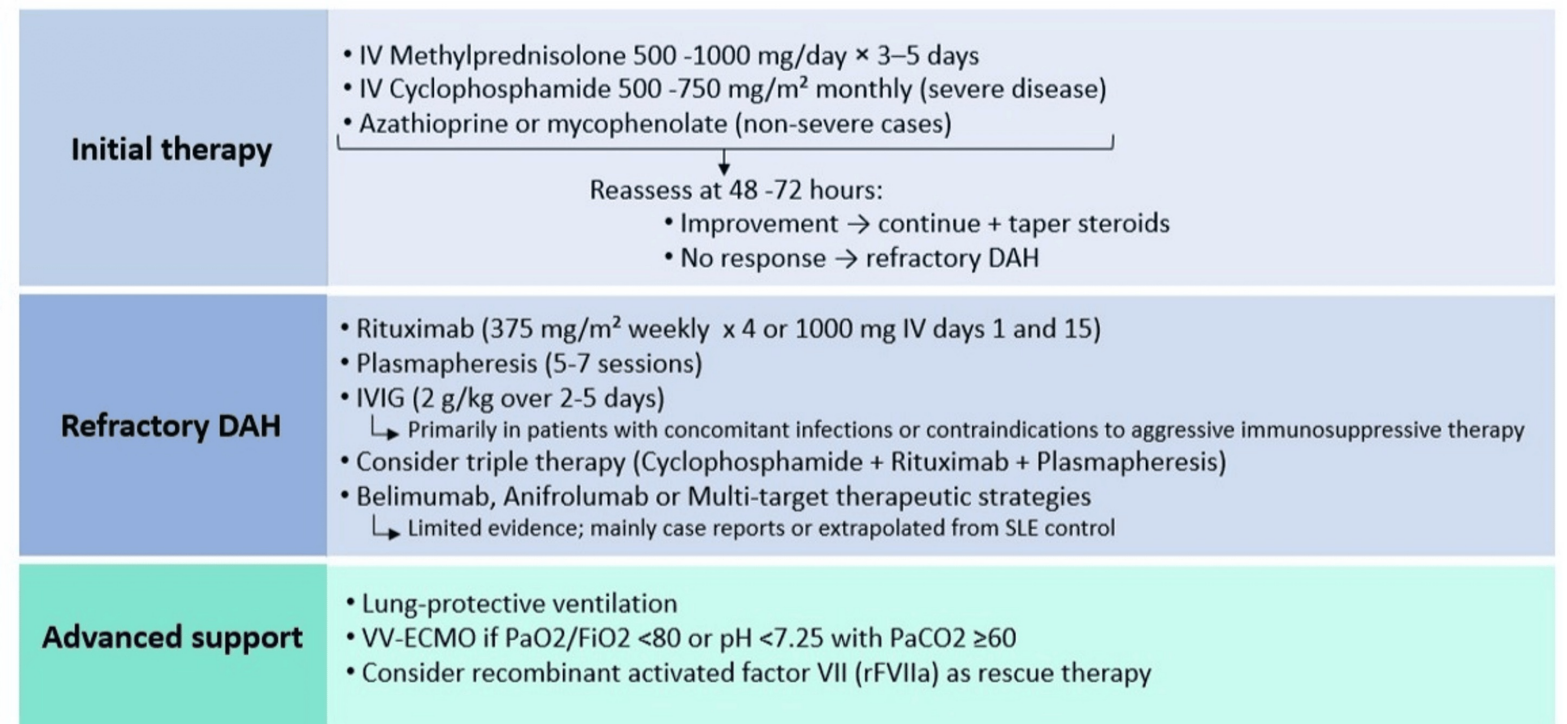
DAH: treatment algorithm. Credit: Image created by the authors. DAH, diffuse alveolar hemorrhage; IV, intravenous; IVIG, intravenous immunoglobulin; PaCO₂, partial pressure of carbon dioxide; PaO₂/FiO₂, ratio of arterial oxygen partial pressure to fractional inspired oxygen; rFVIIa, recombinant activated factor VII; SLE, systemic lupus erythematosus; VV-ECMO, veno-venous extracorporeal membrane oxygenation

Figure 2 summarizes a pragmatic therapeutic algorithm for both initial and refractory DAH management.

Combination therapy in refractory cases

Rituximab has been successfully used in combination with cyclophosphamide, including as part of first-line therapy, and represents an alternative in patients with treatment failure or intolerance to cyclophosphamide, particularly in recurrent DAH [36]. In a case series from China, all patients treated with rituximab survived, with follow-up ranging from 12 to 58 months and no reported relapses [37].

More recently, a 2025 study including seven patients demonstrated that rituximab achieved complete remission without relapse over a mean follow-up of 23 months [38]. Additionally, a multicenter study conducted in Singapore showed that patients receiving triple therapy (cyclophosphamide, rituximab, and plasmapheresis) had a significantly longer median survival (162 days) compared to those treated with plasmapheresis alone (14 days; *P *= 0.0026). The overall remission rate was 74%, with a mean time to remission of 12 days [39].

Multi-target therapeutic strategies have also been reported, combining glucocorticoids, tacrolimus, mycophenolate mofetil, and belimumab in patients refractory to conventional treatment with cyclophosphamide and plasmapheresis [40]. Furthermore, recombinant activated factor VII has been described as a rescue therapy in a case of SLE complicated by refractory pulmonary hemorrhage [41].

Extracorporeal membrane oxygenation in refractory DAH

Extracorporeal membrane oxygenation (ECMO) provides extracorporeal gas exchange by diverting blood from the venous circulation to an external oxygenator and returning it either to the arterial system via the femoral artery (veno-arterial ECMO) or to the venous system via the right internal jugular vein (veno-venous ECMO), the latter offering no direct cardiac support [42].

One of the main limitations of ECMO is the requirement for systemic anticoagulation with intravenous heparin to prevent circuit thrombosis, particularly within the oxygenator, which may increase the risk of ongoing alveolar hemorrhage. However, recent technological advances in extracorporeal systems have enabled reduction-and, in selected cases, even omission-of systemic anticoagulation in this patient population [43].

A systematic review including 38 patients with DAH, of whom 21% had SLE, evaluated the use of ECMO and reported a decannulation survival rate of 94.7%. A significant improvement in the PaO₂/FiO₂ ratio was observed, increasing from 48.2 to 182 mmHg. The 30-day mortality rate was 10.5%, with a mean ECMO duration of 10 days. Veno-venous ECMO was used in 73.7% of cases, representing the most common modality for isolated respiratory support, with no significant differences in outcomes compared to veno-arterial ECMO [44].

Contrary to previous assumptions, heparin was administered in 47.4% of cases, with no significant differences between patients with or without active hemorrhage (*P* = 0.46). More recent series suggest that ECMO with anticoagulation can be safely implemented in DAH using lower anticoagulation targets (activated partial thromboplastin time (aPTT) 40-60 seconds) to minimize bleeding risk [45].

According to the American Thoracic Society guidelines, veno-venous ECMO should be considered in early ARDS (≤7 days) in the presence of severe hypoxemia (PaO₂/FiO₂ <80) or severe hypercapnia (pH <7.25 with PaCO₂ ≥60 mmHg), despite optimal supportive management, including lung-protective ventilation, high positive end-expiratory pressure (PEEP), neuromuscular blockade, and prone positioning, particularly in the context of a potentially reversible etiology [46].

Additional considerations and differential diagnoses

The differential diagnosis of DAH is broad and requires careful evaluation of potential exposures, comorbid conditions, and clinical context. Drug-induced DAH should be considered, particularly in patients with exposure to agents such as amiodarone, abciximab, carbimazole, crack cocaine (including levamisole-adulterated cocaine), nitrofurantoin, leflunomide, penicillamine, propylthiouracil, sirolimus, tumor necrosis factor-alpha inhibitors, or trimellitic anhydride, although drug toxicity accounts for a minority of cases [47].

Other important considerations include conditions associated with acute ARDS, such as COVID-19, as well as complications related to hematopoietic stem cell or lung transplantation and exposure to chemotherapeutic or targeted oncologic therapies. Environmental and inhalational exposures, including vaping and cigarette smoking, have also been implicated, particularly in patients with anti-glomerular basement membrane disease [48-49]. 

Autoimmune causes of DAH include ANCA-associated vasculitis, the most important differential diagnosis, along with anti-glomerular basement membrane disease and antiphospholipid syndrome, which may present as a pulmonary-renal syndrome [50]. The following table summarizes the key differences between DAH associated with SLE and ANCA-associated vasculitis (Table 1).

**Table 1:** Clinical differences between SLE-associated DAH and ANCA-associated vasculitis. ANA, antinuclear antibodies; DAH, diffuse alveolar hemorrhage; SLE, systemic lupus erythematosus; ANCA, antineutrophil cytoplasmic antibodies

Characteristics	SLE-associated DAH	ANCA-associated vasculitis	References
Hemoptysis	46%-50%	Less frequent	[1-3]
Concomitant nephritis	Very frequent (>80%)	Frequent	[3-4]
Age at presentation	Younger (24 ± 11 years)	Variable	[1]
Serology	ANA, anti-dsDNA, anti-Sm, hypocomplementemia	MPO-ANCA or PR3-ANCA (~90%)	[5-6]
Initial presentation	28%-70%	Less common	[1,3]

## Conclusions

DAH represents a severe and life-threatening manifestation of SLE that requires prompt recognition and immediate intervention. Early differentiation from infectious etiologies and other mimickers - particularly ANCA-associated vasculitis - is critical, as delays in appropriate immunosuppressive therapy may significantly worsen outcomes.

High-dose glucocorticoids combined with additional immunosuppressive agents remain the cornerstone of treatment, while adjunctive therapies and ECMO may serve as life-saving strategies in refractory cases.

Despite advances in supportive care and immunomodulatory therapies, mortality remains substantial. Therefore, early diagnosis, timely escalation of therapy, and a multidisciplinary approach are essential to improving survival and clinical outcomes.
